# Label-free quantitative proteomics analysis for type 2 diabetes mellitus early diagnostic marker discovery using data-independent acquisition mass spectrometry (DIA-MS)

**DOI:** 10.1038/s41598-023-48185-3

**Published:** 2023-11-27

**Authors:** Refat M. Nimer, Mahmoud A. Alfaqih, Eman R. Shehabat, Muhammad Mujammami, Anas M. Abdel Rahman

**Affiliations:** 1 Department of Medical Laboratory Sciences, Jordan University of Science and Technology, Irbid, 22110 Jordan; 2 Department of Physiology and Biochemistry, Faculty of Medicine, Jordan University of Science and Technology, Irbid, 22110 Jordan; 3 Department of Biochemistry, College of Medicine and Medical Sciences, Arabian Gulf University, Manama, 15503 Bahrain; 4 Department of Medicine, College of Medicine, King Saud University, 12372 Riyadh, Saudi Arabia; 5 University Diabetes Center, King Saud University Medical City, King Saud University, 12372 Riyadh, Saudi Arabia; 6 Department of Chemistry, Memorial University of Newfoundland, St. John’s, NL A1B 3X7 Canada

**Keywords:** Biochemistry, Diseases

## Abstract

Type-2 diabetes mellitus (T2DM) therapy requires early diagnosis and complication avoidance. Unfortunately, current diagnostic markers do not meet these needs. Data-independent acquisition mass spectrometry (DIA-MS) offers a solution for clinical diagnosis, providing reliable and precise sample quantification. This study utilized DIA-MS to investigate proteomic differential expression in the serum of recently diagnosed T2DM patients. The study conducted a comparative protein expression analysis between healthy and recently diagnosed T2DM groups (discovery cohort). A candidate protein was then validated using enzyme-linked immune assay (ELISA) on serum samples collected from T2DM patients (n = 87) and healthy control (n = 60) (validation cohort). A total of 1074 proteins were identified, and 90 were significantly dysregulated between the two groups, including 32 newly associated with T2DM. Among these proteins, the expression of S100 calcium-binding protein A6 (S100A6) was validated by ELISA. It showed a significant increase in T2DM samples compared to the control group. It was evaluated as a biomarker using the receiver operating characteristic (ROC) curve, consistent with the DIA-MS results. Novel proteins are reported to be involved in the development and progression of T2DM. Further studies are required to investigate the differential expression of candidate marker proteins in a larger population of T2DM patients.

## Introduction

The current opinion refers to Diabetes mellitus (DM) as a range of metabolic diseases characterized by elevated blood glucose levels. DM is a disease of pandemic proportions, and despite worldwide measures to control DM, disease prevalence is still rising. For example, recent estimates published by the International Diabetes Federation demonstrated that the number of individuals with DM is predicted to increase from 537 million in 2021 to 738 million in 2045^1^.

The classification of DM categorizes the disease into type-1 DM (T1DM), caused by the near complete absence of blood insulin, and type-2 DM (T2DM), predominantly affecting obese adults. Although hereditary factors appear to play a stronger role in T2DM etiology than T1DM, recent data demonstrate that the cause of T2DM is complex and multifactorial, with a range of presenting phenotypes^2^.

The complexity of DM could be attributed to several factors. Initially, although an increase in blood glucose is characteristic of all phenotypic presentations of T2DM, it is generally accepted that patients with T2DM also have dysregulation in their lipid and protein metabolism^3,4^. Furthermore, patients with T2DM could have normal blood insulin levels, low blood insulin levels, or insulin resistance accompanied by elevated blood insulin^5^. Moreover, in the late stages of disease progression, beta cell failure could lead to insulin resistance and reduced blood insulin levels. These hormonal and metabolic cues lead to a wide spectrum of disease presentation and phenotype variation, complicating clinical decision-making^6^.

Despite rapid and significant development in DM research over the last few decades, several clinical problems remain to be addressed, especially in biomarker discovery^7^. For instance, currently, available biomarkers do not provide enough power for the early diagnosis or the identification of seemingly healthy individuals at a higher risk of disease development in the future. Moreover, current research indicates several areas for improvement over relying solely on glucose measurements for clinical decision-making in patients with T2DM. Most importantly, using glucose readings alone does not fit well with personalized medicine, where using an algorithm of several variables is more powerful than one^7^.

Considering the above discussion, using an “omics” analytic platform that allows for analyzing many markers could be useful to mend the gaps mentioned above in DM biomarker discovery. In this context, mass-spectrometry (MS) based proteomics is a strong candidate for DM biomarker discovery since it allows for biomarker identification, including the precise and reproducible quantification of their levels in different biological niches^8^. Traditional specific experimental approaches in bottom-up proteomics include data-dependent acquisition (DDA) and data-independent acquisition (DIA)^9^. In a typical DDA-MS experiment, all precursor peptide ions are scanned during the survey scan (MS1) before a predetermined number of precursor ions are selected for further fragmentation (MS2)^10^. While DIA-MS relies on accumulating fragment ions in a defined number of broad isolation windows covering the whole mass-to-charge ratio (m/z) range, allowing for a more comprehensive sample analysis^11^.

DIA-MS analysis is often used due to its high depth of analysis, which yields consistent quantification and extensive proteome coverage^7,12,13^. However, limited studies have used the DIA-MS method for T2DM biomarker discovery in serum samples^4,14,15^.

This investigation utilized the DIA-MS approach to identify and quantitate serum proteins differentially expressed in recently diagnosed T2DM compared with healthy individuals. The findings on how the identified markers highlight differences in biological pathways and processes between the two groups are further discussed.

## Materials and methods

### Clinical sample collection and preparation

Before recruiting patients, the study was approved by King Abdullah University Hospital (KAUH) Institutional Review Board (IRB) (Ref.:9/123/2019). All methods were performed following the KAUH guidelines and regulations. A written informed consent according to the Declaration of Helsinki and institutional approval was obtained from all participants involved in this study. Fasting blood samples were collected from patients recently diagnosed with T2DM (< 3 years) (*n* = 87) who attended the endocrinology and diabetes clinics at KAUH, a tertiary hospital located in the Northern part of Jordan. All patients with T2DM had been diagnosed according to American Diabetes Association guidelines. Patients diagnosed with chronic diseases other than T2DM or with major complications of diabetes were excluded from this study. The Control group involved sixty non-diabetic subjects who were volunteers from Jordan University of Science and Technology (JUST) and their relatives. The age, gender, body mass index (BMI), and ethnic background were matched between the control and T2DM groups, as summarized in Table 1.

**Table 1:** Demographic and clinical characteristics of patients with T2DM and healthy control (HC).

Demographic and clinical characteristics	T2DM (n = 87)		Control (n = 60)		*P*-value
	Mean	SD	Mean	SD	
Age (years)	52.1	9.4	51.1	9.7	0.53
Gender^a^ (Male)	33	NA	23	NA	NA
(Female)	54	NA	37	NA	NA
BMI (kg/m^2^)	30.2	4.9	29.4	4.5	0.28
FBS (mmol/L)	8.0	3.4	5.2	0.4	0.002
HbA1C (%)	7.5	1.2	5.2	0.3	< 0.0001
Total cholesterol (mmol/L)	4.5	2.7	5.2	0.9	0.31
LDL (mmol/L)	2.4	1.0	3.4	0.9	0.001
HDL (mmol/L)	1.2	0.3	1.6	0.5	0.0004
Triglyceride (mmol/L)	2.5	1.3	1.8	0.9	0.04

BMI, body mass index; FBS, fasting blood sugar; HbA1C, hemoglobin A1c; LDL, low-density lipoprotein; HDL, high-density lipoprotein.

*P < 0.05.

^a^Presented as the number of subjects in each group.

10–12 h following an overnight fast, a venous blood sample was collected from each participant, placed in plain tubes, centrifuged at 3000 × g for 10 min, and the serum stored at – 80 °C until further analysis.

### Biochemical measurements

Fasting Blood glucose, total cholesterol, triglycerides, High-Density Lipoprotein (HDL), Low-Density Lipoprotein (LDL), and HbA1c were all measured using a chemical analyzer (Roche Diagnostics, Mannheim, Germany) (Table 1).

### Protein extraction

Serum samples from the T2DM (*n* = 7) and control (*n* = 7) groups (discovery cohort) matched with age, gender, and BMI were used for DIA-MS analysis. SDS-free lysate buffer (7 M urea, 2 M thiourea, and 20 mM Tris–HCl pH) (BGI, China) was added to 100 μL serum sample, and finally, to make up a total volume of 1 mL. The lysate was centrifuged, and the supernatant was collected for protein quantification using a Bradford assay^16^. Quality control of protein extraction and quantification was confirmed by SDS-PAGE (Sodium Dodecyl Sulfate–Polyacrylamide Gel Electrophoresis) (Supplementary Fig. S1).

The reducing agent dithiothreitol (Amresco, Solon, OH, USA) was added to a final concentration of 10 mM and incubated at 37 °C for 30 min, followed by alkylation using iodoacetamide (Sigma, St. Louis, MO, USA) at a final concentration of 55 mM in the dark at room temperature for 45 min. Finally, the mixture was centrifuged at 25,000 g for 20 min at 4 °C. The mixture of proteins would be passed through a solid phase extraction (SPE) C18s (Agela Technologies, China) column for protein enrichment. Finally, 75% ACN was used to elute lower-abundance proteins^17^.

### In-solution protein tryptic digestion

Enzymatic hydrolysis of proteins in solution was performed by mixing 100 µg of proteins with 50 mM NH_4_HCO_3_ by 4 times volumes. A 2.5 μg trypsin (Hualishi Scientific, China) at a 40:1 ratio was added to samples and then incubated for 4 h at 37 °C. Finally, the resulting peptides were desalted with a Strata × column (Phenomenex, USA) and vacuumed till dryness.

### DDA and DIA analysis by nano-LC–MS/MS

The dried peptide samples were reconstituted with mobile phase A (2% ACN, 0.1% FA), centrifuged at 20,000 g for 10 min, and the supernatant was taken for injection. Separation was carried out by a nano C18 column (150 μm internal diameter, 1.8 μm particle size, 35 cm column length) coupled in Thermo UltiMate 3000 UHPLC liquid chromatograph (Thermo Scientific, USA) at a flow rate of 500 nL/min by the following effective gradient: 0–5 min, 5% mobile phase B (98% ACN, 0.1% FA); 5–120 min, mobile phase B linearly increased from 5 to 25%; 120–160 min, mobile phase B rose from 25 to 35%; 160–170 min, mobile phase B rose from 35 to 80%; 170–175 min, 80% mobile phase B; 175–180 min, 5% mobile phase B. The nanoliter liquid phase separation end was directly connected to the mass spectrometer in the following settings.

For DDA (data-dependent acquisition) analysis, LC-separated peptides were ionized by nanoESI. They injected into tandem mass spectrometer Q-Exactive HF X (Thermo Fisher Scientific, San Jose, CA) with DDA detection mode. The main settings were ion source voltage 1.9 kV; MS scan range 350–1500 m/z; MS resolution 120,000, maximal injection time (MIT) 100ms; MS/MS collision type HCD, collision energy NCE 28; MS/MS resolution 30,000, MIT 100ms, dynamic exclusion duration 30 s. The start m/z for MS/MS was fixed to 100. Precursor for MS/MS scan satisfied: charge range 2+ to 6+, top 20 precursors with intensity over 5E4.AGC was: MS 3E6, MS/MS 1E^5^.

For DIA (data independent analysis), the main settings were ion source voltage 1.9 kV; MS scan range 400–1250 m/z; MS resolution 120,000, MIT 50 ms; 400–1250 m/z was equally divided to 45 continuous windows MS/MS scan. MS/MS collision type HCD, MIT was auto mode. Fragment ions were scanned in Orbitrap, MS/MS resolution 30,000. The collision energy was distributed mode: 22.5, 25, 27.5, AGC was 1E^6^.

### Data analysis

The DDA sample data generated by the Q Exactive HF mass spectrometer was processed using the MaxQuant software (v. 1.5.3.30, Max Planck Institutes, GER), incorporating the Andromeda search engine. This enabled us to analyze and identify the spectra. To generate a spectral library, we utilized Spectronaut software (v. 13.12.200217.43655, Biogonosys, USA) in conjunction with the processed data^18,19^.

Several parameters were employed during the MaxQuant data analysis. The enzyme used for digestion was trypsin, and peptides with a minimum length of 7 amino acids were considered. To ensure accurate identification, a minimum of 1 unique peptide was required. The false discovery rate (FDR) at the peptide-spectrum match (PSM) level and protein level was set at 0.01. Additionally, fixed modifications included carbamidomethyl (cysteine), while variable modifications encompassed oxidation (methionine) and acetylation (protein N-terminus). The database used for protein sequence matching was UniProt homo_ (172419 sequences). The protein sequences retrieved were obtained from the UniProt database, accessible at https://www.uniprot.org/. To ensure accurate retention time calibration of the DIA data, iRT peptides (Biognosys, Switzerland) were utilized. Subsequently, employing the target-decoy model for Sequential Window Acquisition of all Theoretical Mass Spectra (SWATH)-MS, a false positive control was implemented with a 1% false discovery rate (FDR), yielding reliable quantitative outcomes. The subsequent steps encompassed protein quantification, data preprocessing, and significant differential analysis, which were carried out using MSstate software^20^.

The differential analysis relied on a linear mixed-effect model to calculate fold change values. The data was preprocessed according to the predefined comparison group, and significance testing was conducted based on the established model. Following this, differential protein screening was performed using a fold change threshold of > 1.5 and a significance criterion of a *P* value < 0.05. These parameters were employed to identify proteins displaying significant differences in expression levels.

In order to determine potential biomarkers multivariate analysis, partial least-square discrimination analysis (PLS-DA) was conducted in MetaboAnalyst Software V5 (Montreal, QC, Canada) (http://www.metaboanalyst.ca). Additionally, potential biomarkers were evaluated by performing the receiver operating characteristic ROC curve analysis.

The functional classification of differentially expressed proteins (DEPs) and functional enrichment analysis were performed using Gene Ontology (GO) (http://www.geneontology.org) and Kyoto Encyclopedia of Genes and Genomes (KEGG) databases (http://www.genome.jp/kegg/), respectively. Moreover, EuKaryotic Orthologous Groups (KOG) were applied to classify protein orthologs. Protein–protein interaction (PPI) and subcellular localization analysis of the DEPs were performed using the Search Tool for the Retrieval of Interacting Genes (STRING) v11.5 database (https://string-db.org/) and Blast2go software (www.blast2go.com), respectively^21, 22^.

### Enzyme-linked immunosorbent assay (ELISA)

A protein S100A6 identified by LC–MS/MS was selected for further analysis by ELISA.

The levels of S100A6 in blood samples from the validation cohort group (control group, n = 60; and the T2DM group, n = 87) were determined quantitatively using the S100A6 ELISA Kit (CSB-E13089h, Cusabio, PRC); Cambridge, UK), following the instructions provided by the manufacturer.

### Statistical analysis

The student’s t-test was used to compare the two groups and establish the statistical significance of the results. The threshold for statistical significance was set at *P* 0.05. The receiver operating characteristic curves (ROC) were generated using GraphPad Prism program v 8.0.

## Results

### Characteristics of patients and healthy control (HC)

The baseline characteristics of the study group are presented in Table 1. The mean age of the T2DM and HC groups was 52.1 ± 9.4, and 51.1 ± 9.7 years, respectively. The two study groups had no significant differences in age, BMI, and total cholesterol (*P* > 0.05). However, LDL cholesterol and HDL cholesterol were significantly (*P* < 0.05) higher in HC (3.4 ± 0.9 and 1.6 ± 0.5, respectively) than T2DM group (2.4 ± 1.0 and 1.2 ± 0.3, respectively).

As expected, FBS and HbA1c were significantly higher in patients with T2DM (8.0 ± 3.4 and 7.5 ± 1.2, respectively) than HC group (5.2 ± 0.4 and 5.2 ± 0.3, respectively) (*P* < 0.05). Moreover, triglyceride was significantly (*P* < 0.05) higher in T2DM (2.5 ± 1.3) than in the HC group (1.8 ± 0.9).

### Identification of differentially expressed proteins in T2DM compared with normal serum

In this project, Q-Exactive HF X (Thermo Fisher Scientific, San Jose, CA) was used to acquire mass spectrometry (MS) data for 14 samples (7 patients and 7 controls) in DIA mode, 1074 proteins were identified, of which 90 DEPs were detected. Supplementary Table S1 shows 41 DEPs were upregulated, and 49 DEPs were downregulated in the serum from the T2DM group compared with the HC group.

**Figure 1:**
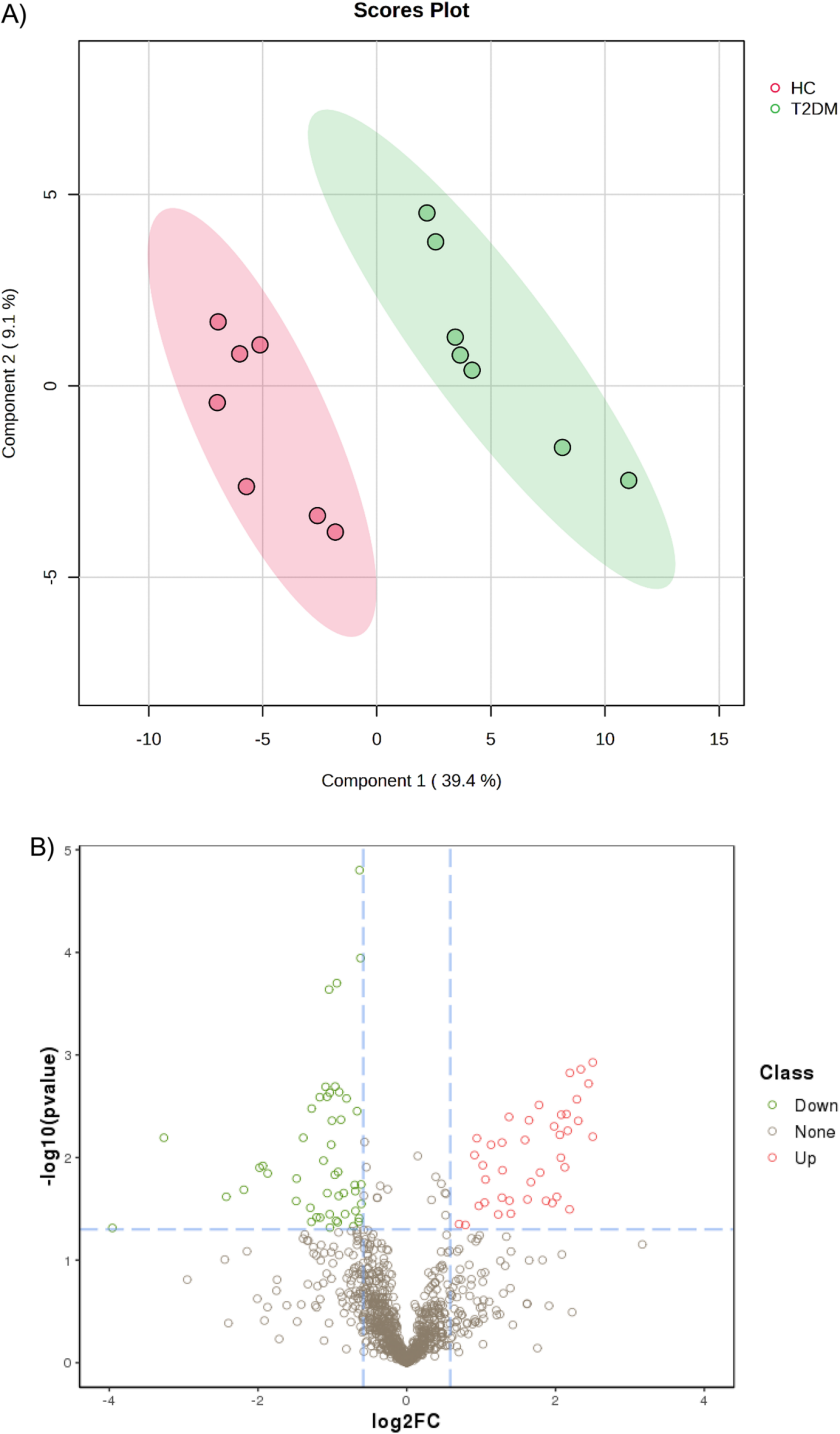
(**A**) Partial Least-Squares Discriminant Analysis (PLS-DA) of T2DM and healthy serum proteomics data. (**B**) Volcano plot of differentially expressed proteins (DEPs) in serum of patients with T2DM and control. In this volcano plot, red dots represent proteins with a significant fold change (FC) > 1.5; green dots proteins with a significant FC < 0.67; grey dots proteins with no significant change.

A PLS-DA model was constructed to investigate and analyze the separation of the T2DM and healthy groups. As shown in Fig. 1A, the T2DM and healthy groups were separated. The first and second principal components (PC1 and PC2) explained 39.4 and 9.1% of the variation in samples in the PLS-DA score plot.

The volcano plot in Fig. 1B depicted differential abundances (T2DM versus control), with the log2 ratio on the x-axis representing the fold change and the − log10 (p-value) on the y-axis depicting significance. A horizontal line represents the position of a *P*-value of 0.05, and the positions of the upper right (Fold change > 1.5) and upper left (fold change < 0.67) are represented by two vertical lines. The red and green dots indicate up-regulated and down-regulated proteins, respectively. Ninety serum proteins were significantly altered, with 41 being upregulated and 49 being downregulated.

### Evaluation of biomarkers between T2DM and HC

**Figure 2:**
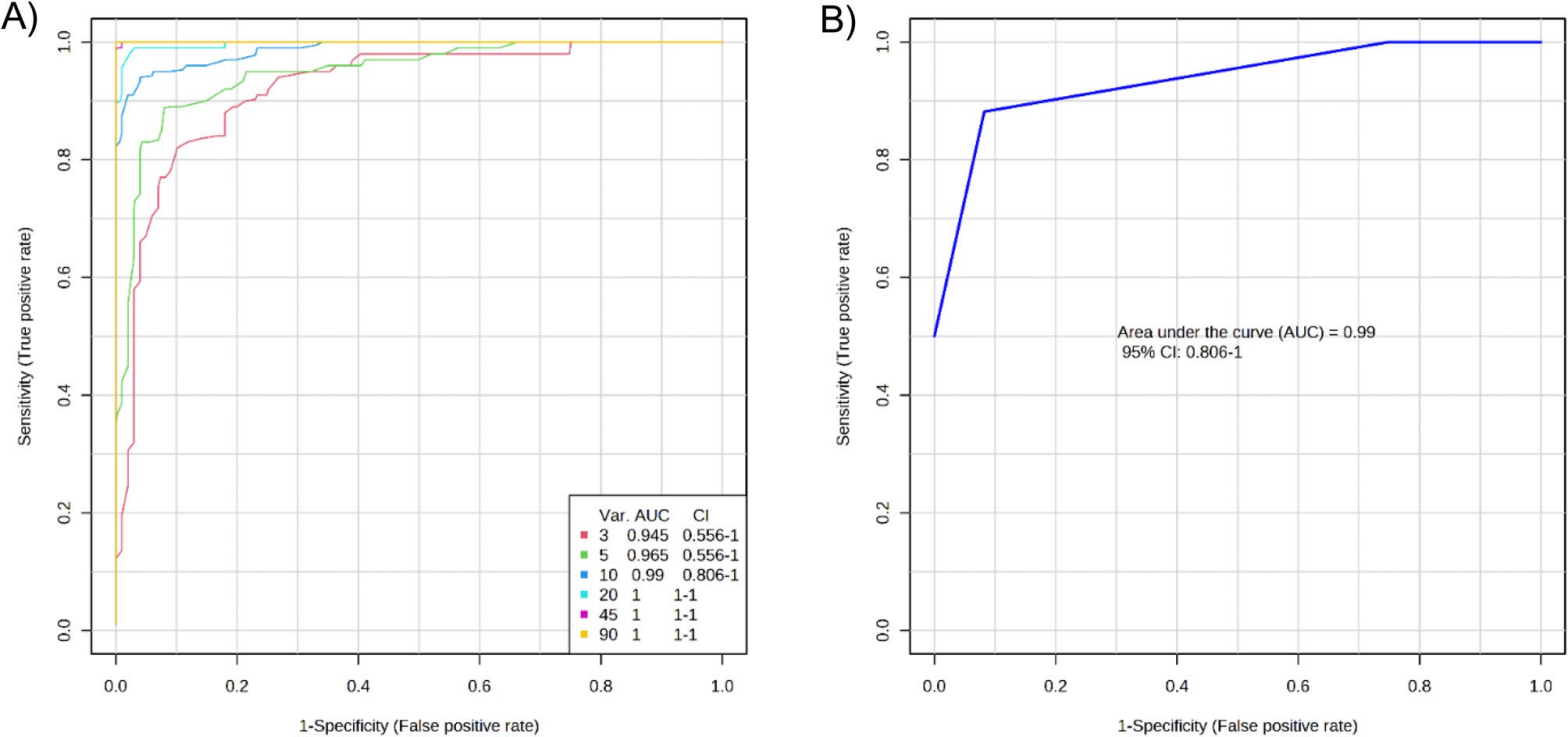
Biomarker prediction by Multivariate ROC curve based exploratory analysis. (**A**) An Overview of all ROC curves created by MetaboAnalyst 5.0 from 6 different biomarker models considering the different number of features (3, 5,10, 20, 45, and 90) with their corresponding AUC value and confidence interval. (**B**) ROC curve for selected biomarker model 3.

Based on the significant DEPs in the T2DM and HC groups, a multivariate exploratory ROC analysis was conducted utilizing PLS-DA as a classifier and feature ranker (Fig. 2A). For the most important 10 proteins (alpha-2-HS-glycoprotein, epididymis luminal protein 213, Ig heavy chain variable region, anti-thrombopoietin receptor single-chain variable fragment, IBM-A1 heavy chain variable region, glutaminyl-peptide cyclotransferase, 10E8 heavy chain variable region, olfactory receptor 4D6, alpha-globin, and leucine-rich repeat-containing protein 4C) the AUC of the exploratory ROC curve was 0.99 (Fig. 2B).

### Functional classification and annotation of DEPs

#### GO enrichment analysis

**Figure 3:**
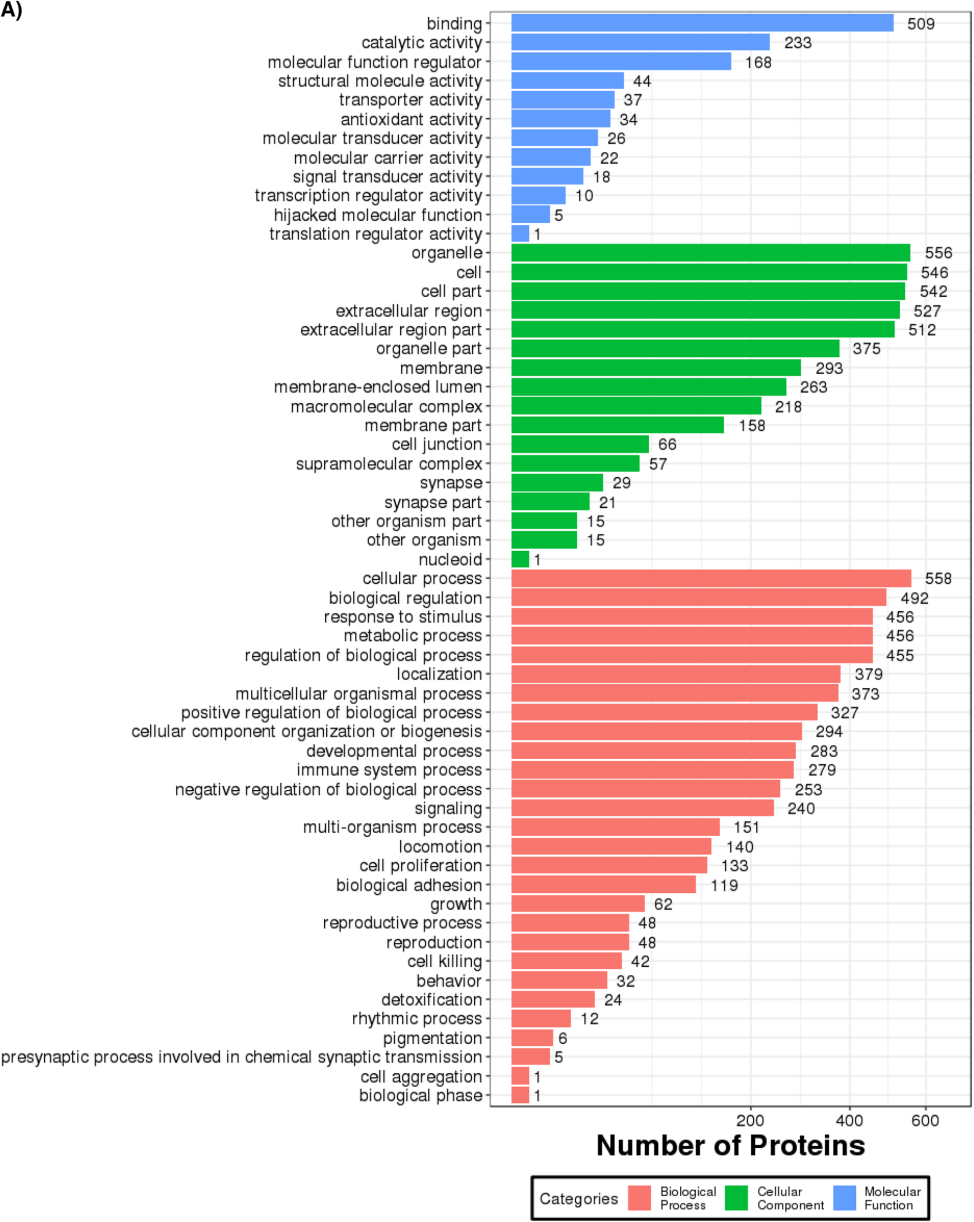

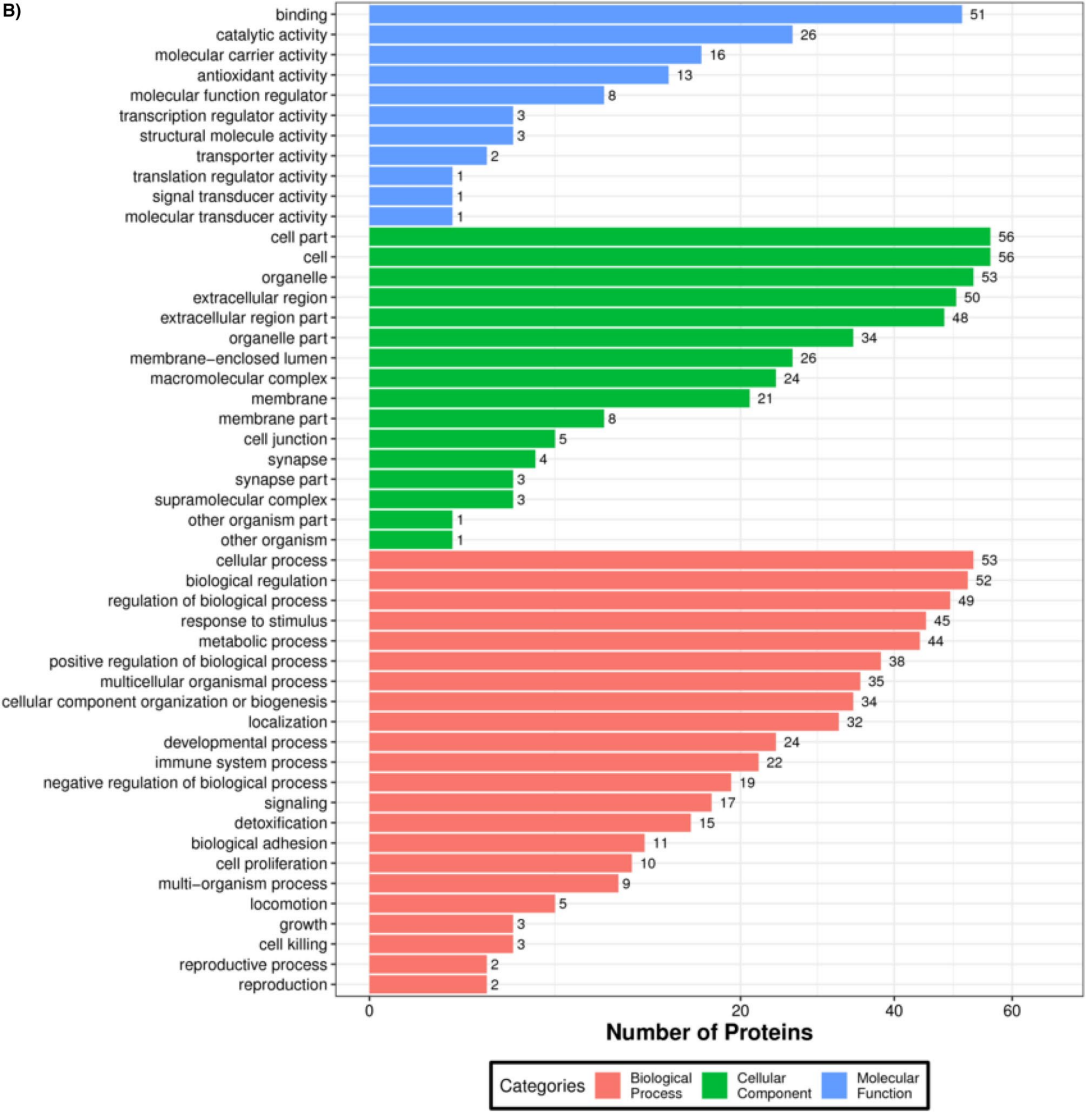

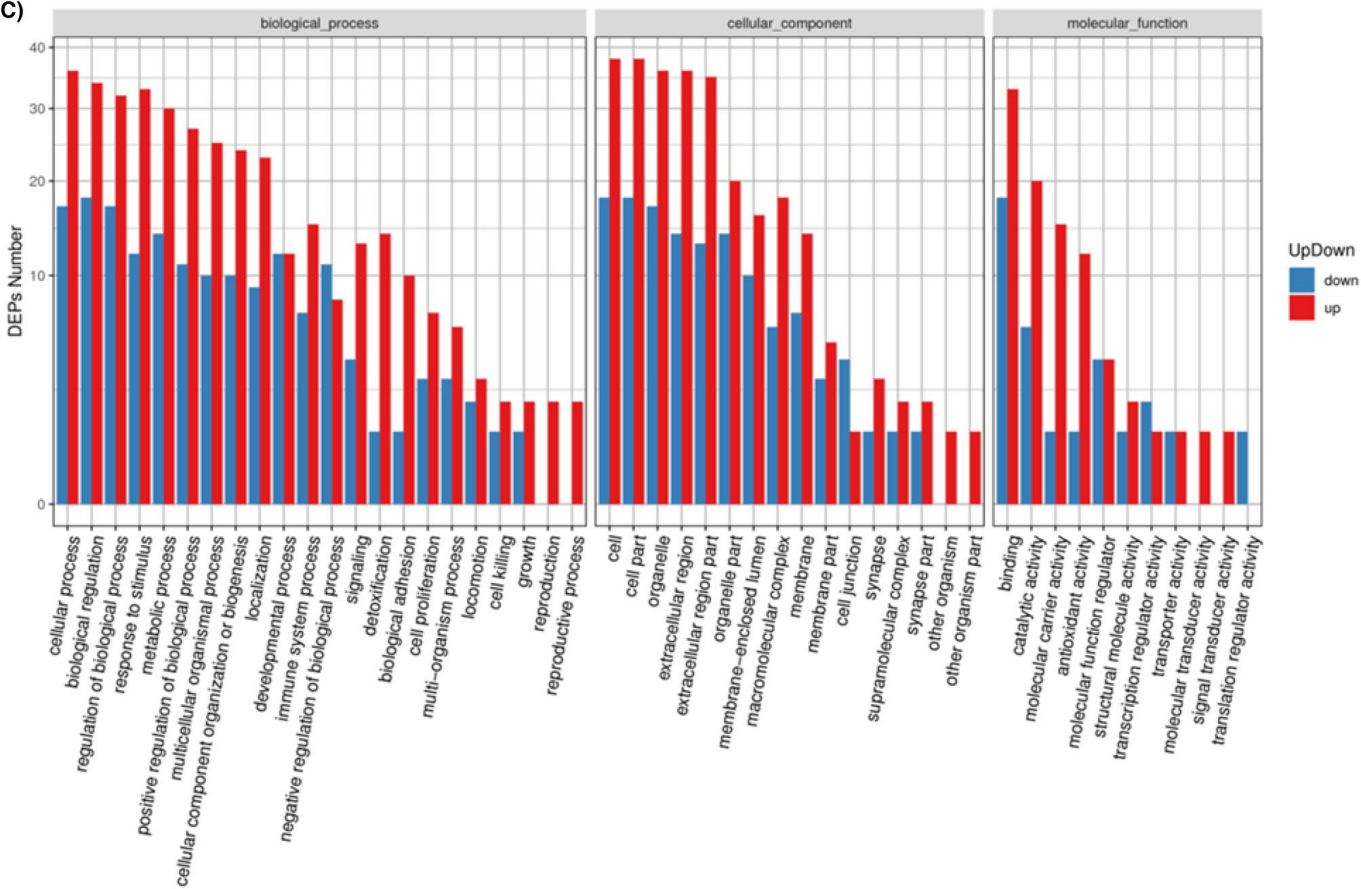
(**A**) Functional GO classification of all the identified serum proteins in T2DM. (**B**) Differential protein function classification. The X-axis represents the number of differential proteins, and the Y-axis represents the GO annotation entry. (**C**) Up or down-regulation of differential proteins in GO function classification. The X-axis represents the GO annotation entry, and the Y-axis represents the number of differential proteins with up or downregulation.

A GO annotation study was carried out using the Blast2GO software to determine the functional significance of all identified proteins in the serum of T2DM. Figure 3A includes protein information and results visualization. The most enriched biological processes (out of 28 GO terms) were: ‘cellular process,’ ‘biological regulation,’ ‘response to stimulus,’ ‘metabolic process,’ and ‘regulation of the biological process,’ and. The most enriched cell components (out of 17 GO terms) were: ‘organelle,’ ‘cell,’ and ‘cell part.’ The most enriched molecular functions (out of 12 GO items) were: ‘binding,’ ‘catalytic activity, and ‘molecular function regulator.’

Next, DEPs were subjected to GO enrichment analysis (Fig. 3B). GO analysis and annotation classified DEPs into biological processes, cellular components, and molecular functions (Fig. 3B). Cells, parts, and organelles were the most abundant in the cellular component category (Fig. 3B). The top 5 biological processes were cellular process, biological regulation, regulation of the biological process, response to stimulus, and metabolic process (Fig. 3B). The top 5 molecular functions were binding, catalytic activity, carrier activity, antioxidant activity, and molecular function regulator (Fig. 3B).

The GO functional annotation results of the DEPs between patients of T2DM and control are shown in Fig. 3C. For the cellular component domain, 56 DEPs were mainly concentrated in the cell part, among which the top upregulated and downregulated proteins were hemoglobin subunit alpha, and hemoglobin beta chain, respectively. For the molecular function domain, 51 DEPs were mainly in binding; among the top, upregulated proteins were the S100A6 protein. For the biological process domain, the DEPS were primarily involved in cellular processes (53 DEPs) and biological regulation (30 DEPs), and notably, the S100A6 protein was among the most upregulated proteins.

### KOG annotation of DEPs

**Figure 4:**
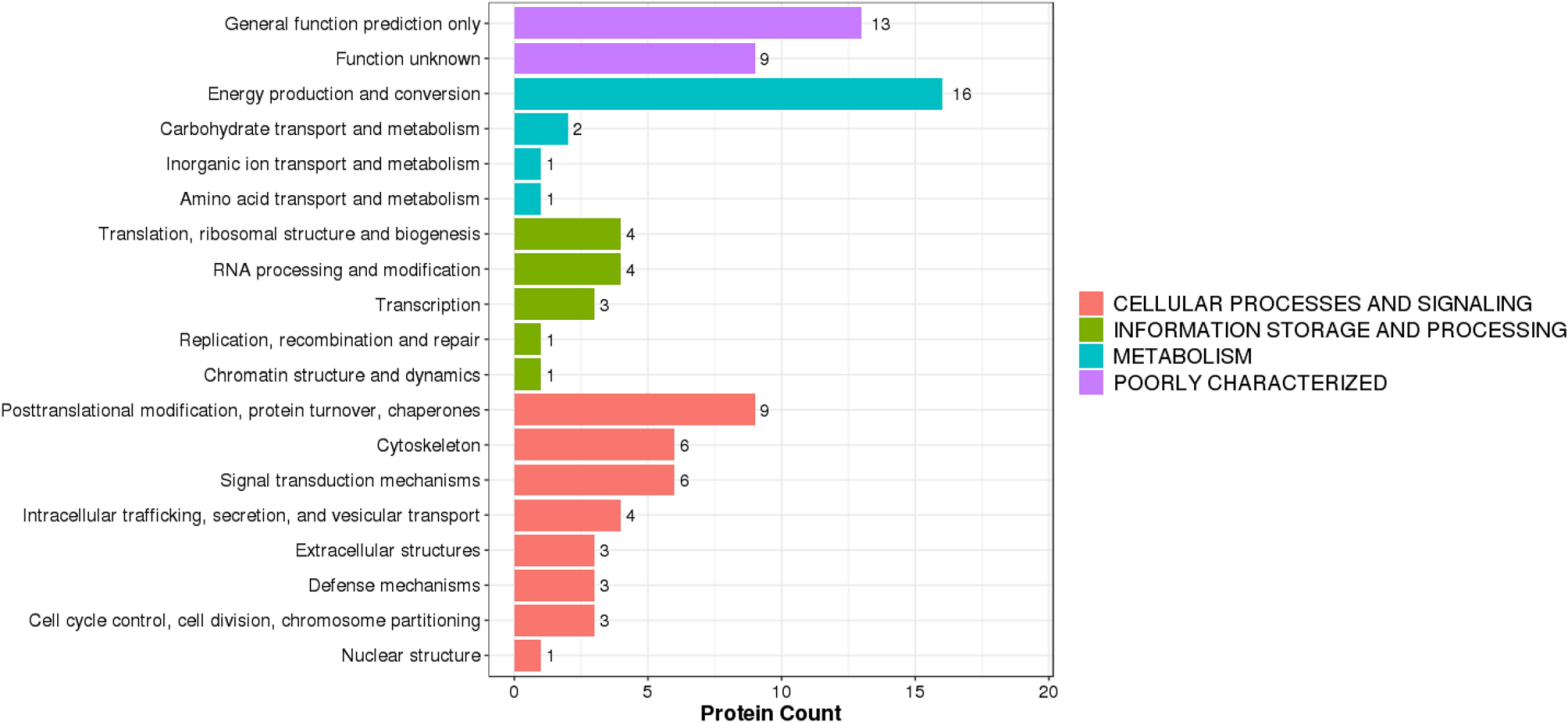
KOG annotation of DEPs. X-axis displays the DEPs count; Y-axis displays KOG terms.

For DEPs, their annotated KOG terms were extracted and represented as bar plots in Fig. 4. According to the KOG study, most annotated DEPs may impact “cell processes and signaling.” In this regard, most DEPs were associated with post-translational modification, protein turnover, chaperone activity, cytoskeleton, and signal transduction mechanisms.

### Pathway enrichment analysis of DEPs

It is possible to get a deeper understanding of the biological roles of the DEPs via analyses based on metabolic pathways. Therefore, in the present study, the KEGG pathway annotated the DEPs identified in the serum samples from patients with T2DM and control.

**Figure 5:**
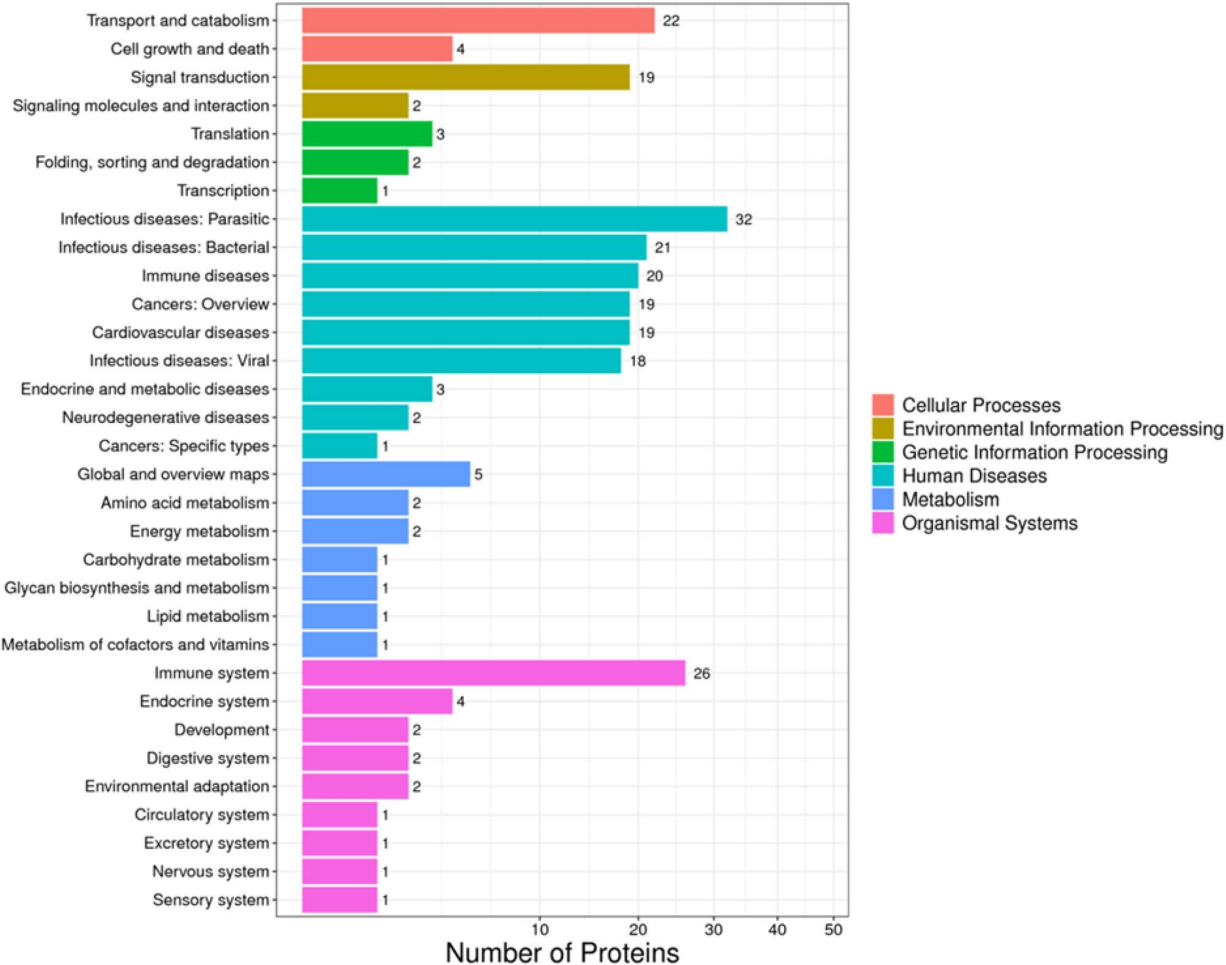
Differential protein pathway classification. The X-axis represents the number of differential proteins, and the Y-axis represents the pathway annotation entry.

The KEGG-enriched pathways DEPs up- and down-regulated clusters are presented in Fig. 3. The most enriched pathways were as follows: infectious diseases (32), immune system (26), transport and catabolism (22), and signal transduction (19) (Fig. 5).

### Subcellular localization and protein–protein interaction network analyses of DEPs

**Figure 6:**
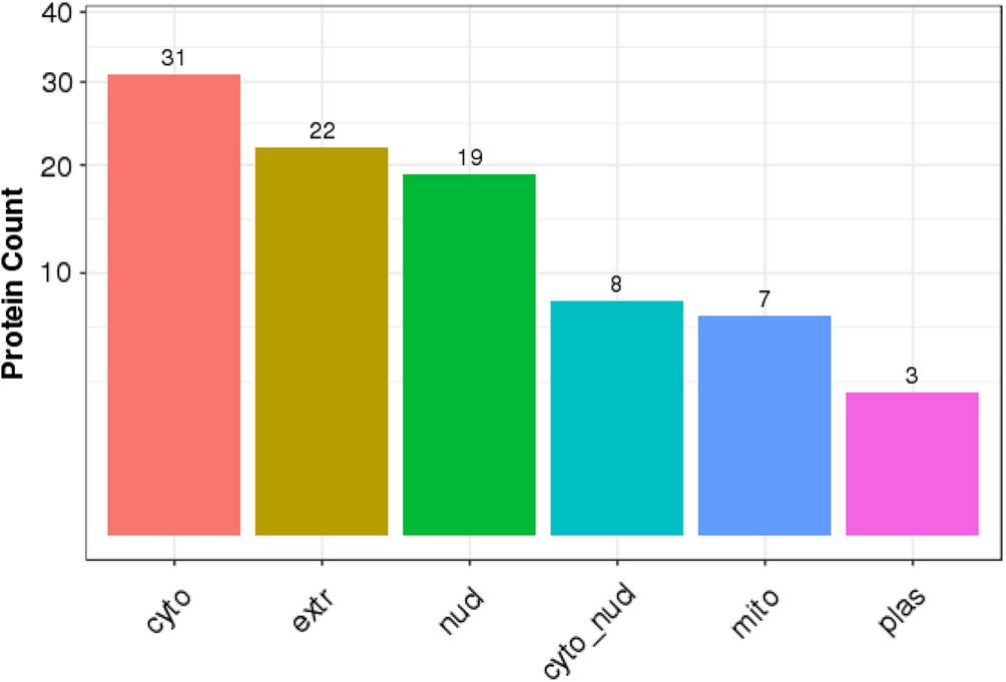
Bar chart of subcellular localization. X-axis represents the subcellular structure term; Y-axis represents protein count.

Analysis of subcellular localization of the identified DEPs using WoLF PSORT software (https://wolfpsort.hgc.jp/)^23^ showed that most proteins (32) were localized in the cytoplasm. The next most prominent localization was extracellular proteins (22), followed by the nucleus (19) (Fig. 6).

DEPs were widely distributed in the nucleus, cytoplasm, plasma membrane, and extracellular space (Fig. 6).

**Figure 7:**
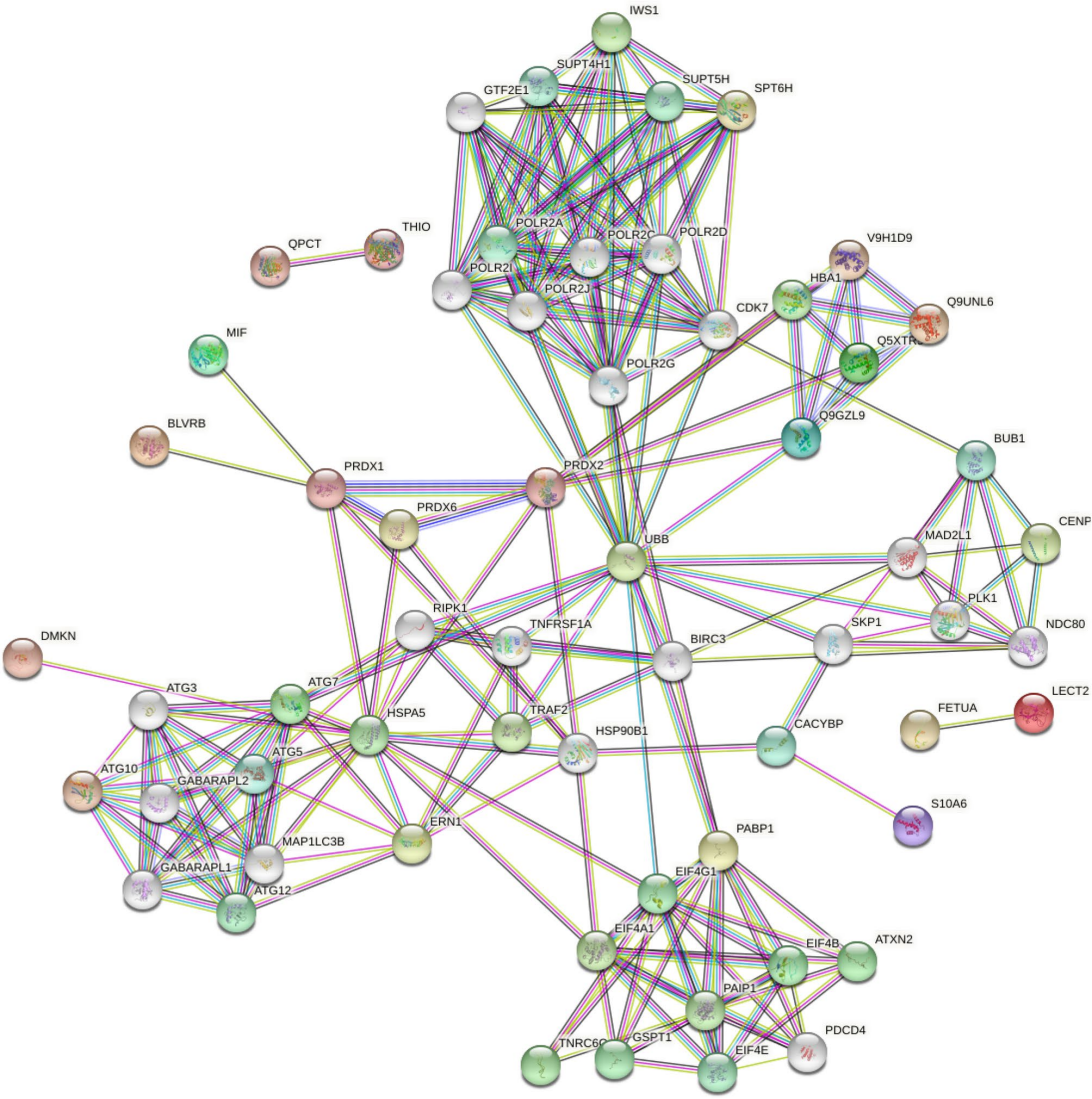
Protein–Protein Interaction (PPI) network of DEPs. Colored balls represent individual proteins, while lines show interactions between proteins.

To better understand the possible protein–protein interaction (PPI) of the DEPs, we performed PPI proteomics network analysis utilizing the STRING) database (Fig. 7). The PPI network was produced when the median confidence level of 0.4 was used.

### Serum levels of S100A6 by validation experiment

To validate the DEPs in the LC–MS/MS experiment, S100A6 was selected for validation by ELISA due to the novelty of this candidate biomarker identified during proteomics screening.

**Figure 8:**
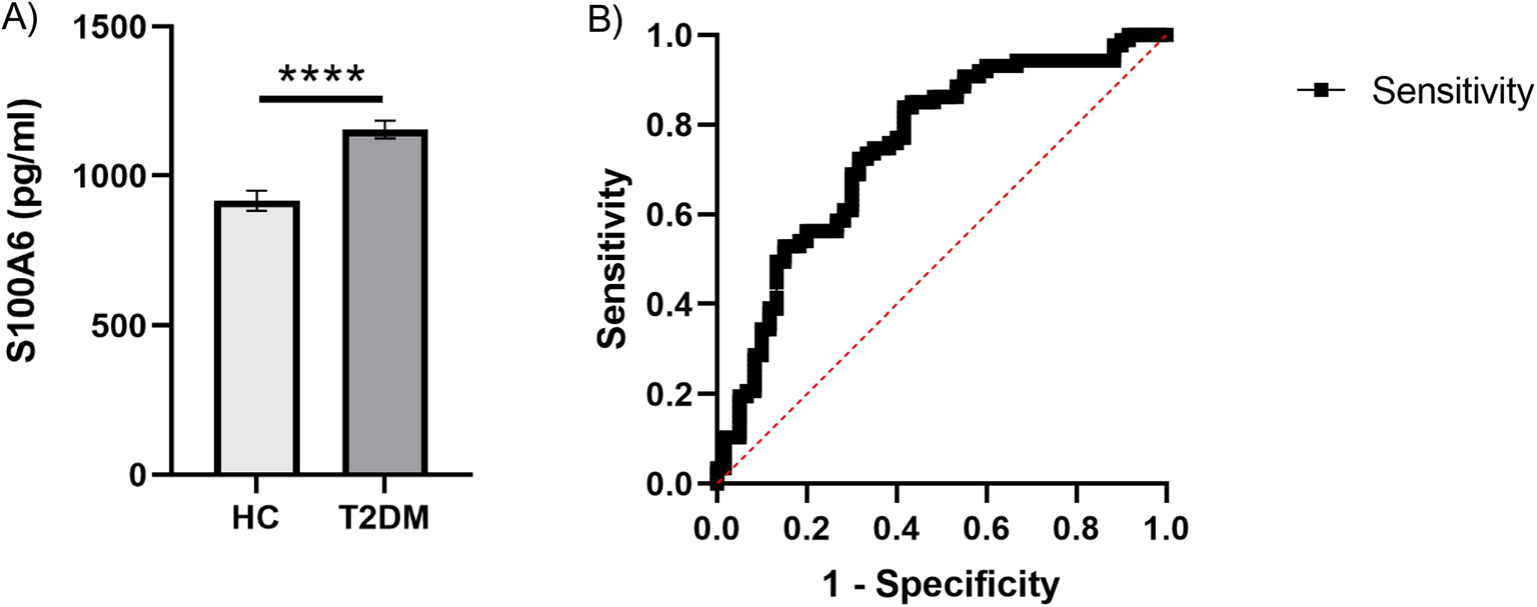
(**A**) S100A6 protein concentration in serum. The horizontal axis represents the control group (n = 60) and the T2DM group (n = 87). The y-axis indicates the concentration of S100A6. Values are shown as the mean ± SEM; *P < 0.05. (**B**) ROC curve analysis of validated differentially expressed protein (S100A6). ROC‑AUC of S100A6 was 0.7487 (0.6668 to 0.8305, *P* < 0.0001).

Our data also showed a significant increase in the S100A6 levels in the T2DM group compared to the control group (Fig. 8A). Therefore, the result is consistent with the LC–MS/MS experiment data. Furthermore, sensitivity and specificity for S100A6 as a biomarker were accessed by receiver operating characteristic (ROC) analysis. The area under the curve (AUC) for S100A6 (AUC = 0.7487, 95% confidence interval (CI): 0.6668 to 0.8305, *P* < 0.0001) (Fig. 8B).

## Discussion

Molecular biomarkers for T2DM are still desperately needed because of the multifactorial and multigenetic nature of the disease, as well as several clinical challenges that remain to be solved. Finding novel diagnostic biomarkers with the use of mass spectrometry (MS)-based proteomics could provide a solution to the clinical issues. In the present study, we aimed to demonstrate alterations in the serum proteome in patients recently diagnosed with T2DM using DIA-MS-based proteomics.

Several proteomic studies have been conducted in the context of T2DM research^24,25^. However, very few studies use DIA-MS to comprehensively analyze the proteome in the serum of T2DM patients^4^.

We identified a significant decrease in the level of abundance of 49 proteins in the serum of T2DM compared to the healthy group, while 41 proteins were increased significantly. Among 49 downregulated proteins, 15 proteins (Centrosomal protein of 290 kDa, zinc finger BED domain-containing protein 1, polyadenylate-binding protein 1, centromere protein F, 40S ribosomal protein S8, defensin-5, leiomodin-3, glutaminyl-peptide cyclotransferase, beta-taxilin, secretoglobin family 3A member 1, ubiquitin-like-conjugating enzyme ATG10, zinc finger SWIM domain-containing protein 6, protein kinase C and casein kinase substrate in neurons protein 2, integral membrane protein 2B, and UHRF1-binding protein 1) have not been previously reported in direct association with T2DM. Whereas in up-regulated proteins, 17 proteins (NEDD4-binding protein 3, serine/threonine-protein kinase/endoribonuclease IRE1, hemoglobin subunit delta, protein S100-A6, polyubiquitin-B, protein S100-A4, D-dopachrome decarboxylase, neurotensin/neuromedin N, chronic lymphocytic leukemia up-regulated 1 opposite strand Dermokine, Coiled-coil domain-containing protein 80, Transcription elongation factor SPT6, Olfactory receptor 4D6, Beta-globin Showa Yakushiji variant, Hemoglobin subunit delta, Beta-globin, Hemoglobin delta-beta fusion protein, and HCG1745306, isoform CRA_a) were considered novel observation in the context of T2DM.

Some DEPs, such as peroxiredoxin isoforms (1, 2, and 6), natriuretic peptides A, and complement protein C4B were previously studied in patients with T2DM^26–28^. Moreover, various isozymes of carbonic anhydrase play a role in T2DM, which fits well with our findings^29^.

Proteomic analysis has shown upregulated macrophage migration inhibitory factor (MIF) expression levels in T2DM patients. MIF is an inflammatory cytokine derived from T-cells^30^. Several studies have demonstrated that serum levels of MIF were elevated in T2DM and its complications^31,32^.

Furthermore, thioredoxin was upregulated in the serum of patients with T2DM. Thioredoxin is a marker of oxidative stress, and glucose intolerance was associated with high levels of thioredoxin^33^.

One of our findings was that the immunological class of proteins was extensively represented and significantly up or down-regulated in T2DM. Most of these proteins include the Ig heavy and light chains (κ, λ). Since immunity may contribute to T2DM pathogenesis^34^ and, on the other hand, reduction of immunity is one of the principal consequences of T2DM, the function of immunity in T2DM is heterogeneous^35^.

Furthermore, Hb chains (Hb subunits α, β, γ, and δ) were upregulated in T2DM compared with the control. Only the Hb subunit α had been previously identified as a biomarker for T2DM among the aforementioned Hb-associated proteins^36^.

Analysis of subcellular locations showed that the DEPs were mainly in the cytoplasm, extracellular, and nucleus. As expected, the proteins that have a role in the pathogenesis of T2DM are localized primarily in the nucleus and cytoplasm^37^. Additionally, the extracellular localization is consistent with that increased extracellular matrix (ECM) protein synthesis is a hallmark of all long-term diabetes problems^38^.

Among these, an interesting protein identified in our study was the S100A6 protein. It belongs to the S100 family of calcium-binding proteins, which function in Ca2+-dependent protein–protein interactions (PPIs) that mediate intracellular and intercellular cellular regulation^39^. However, the actual biological or cellular function of S100A6 is not fully known, and conflicting functions have been hypothesized^40^.

The direct association of circulatory S100A6 protein with T2DM was not reported before in the literature to the best of our knowledge. Therefore, the S100A6 protein was examined by ELISA in a relatively larger sample size from the same sample setting to verify the LC–MS/MS findings. The findings revealed a significant association between increased S100A6 protein and T2DM. According to the ROC analysis, S100A6 can serve as a potential diagnostic biomarker for T2DM.

The experiments at the genome level showed that high glucose-stimulated S100A6 transcription by binding of c-Myc to its promoter to control S100A6 expression directly^41^. Using cumulus cells from mice, Jiang et al. found that S100A6 was significantly increased in the induced diabetes animals compared to the control group^42^. The S100A6 is a hepatocyte that contributes to hepato-pancreatic communication to reduce insulin production and promote the progression of T2DM in individuals with nonalcoholic fatty liver disease^43^. S100-A6 may be involved in the signal transduction of Ca2+-induced insulin release from pancreatic B cells, suggesting that it plays a role in the insulin release process^44^.

Despite the small number of patients, the study's strength lies in the fact our study is one of the few papers to identify proteomics changes in the serum of recently diagnosed T2DM using DIA-MS. Additionally, some DEPs may potentially elucidate underlying disease mechanisms' diagnostic and prognostic indicators; however, no further verification study was undertaken to verify the link between clinical features and the proteomics data, which should be clarified for the development and implementation of clinical practice. Notably, the other novel proteins will be investigated in the future using a high sample size. Certainly, our results represent the starting point for more in‐depth studies.

In conclusion, we applied a proteomic technique with label-free DIA-MS analysis to find alterations in protein profiles in the serum of recently diagnosed T2DM patients and healthy controls. We found proteins whose expression levels were altered in the serum of recently diagnosed T2DM patients compared to HCs. To our knowledge, our data showed the overexpression levels of S100A6 protein in the serum of recently diagnosed T2DM patients for the first time. This suggests that S100A6 may have a critical role in the pathogenesis of T2DM. Furthermore, our results revealed other DEPs identified herein had not previously been reported in T2DM, such as NEDD4-binding protein 3, neurotensin/neuromedin N, and beta-taxilin. As a result, this proteomics study provides reference proteins for future research on T2DM and may help develop treatments and meet the goals in early diagnosis and complication avoidance of T2DM. However, these findings need to be further studied and validated in future studies.

### Supplementary Information

Supplementary Information.

## Data Availability

The datasets used and/or analyzed during the current study are available from the corresponding author, Refat Nimer, on reasonable request.
