# Supplementary material for: Label-free quantitative proteomics analysis for type 2 diabetes mellitus early diagnostic marker discovery using data-independent acquisition mass spectrometry (DIA-MS)
Source: Sci Rep. 2023 Nov 27;13:20880. doi: 10.1038/s41598-023-48185-3 (PMC10682489; doi:10.1038/s41598-023-48185-3)
Supplement: Supplementary file 1 — Supplementary Information. [file 41598_2023_48185_MOESM1_ESM.docx]

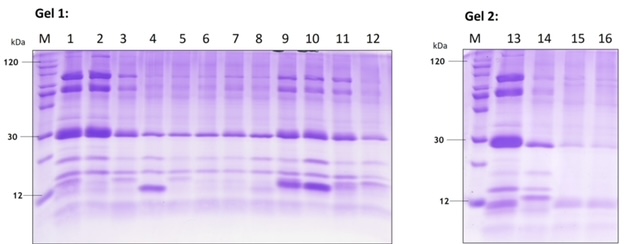


**Fig.1. Quality Control of Protein Extraction and Quantification through SDS-PAGE Analysis:** SDS-PAGE gels, denoted as Gel 1 and Gel 2, were employed to verify the quality control of protein extraction and quantification. Gel 1 and Gel 2 originate from the same experimental setup and were concurrently processed. The gel composition is as follows:

Lane M: Molecular weight markers

Lanes 1-6: Serum samples from the control group

Lanes 7-14: Serum samples from patients diagnosed with Type 2 Diabetes Mellitus (T2DM)

Lanes 15-16: Quality assurance standard


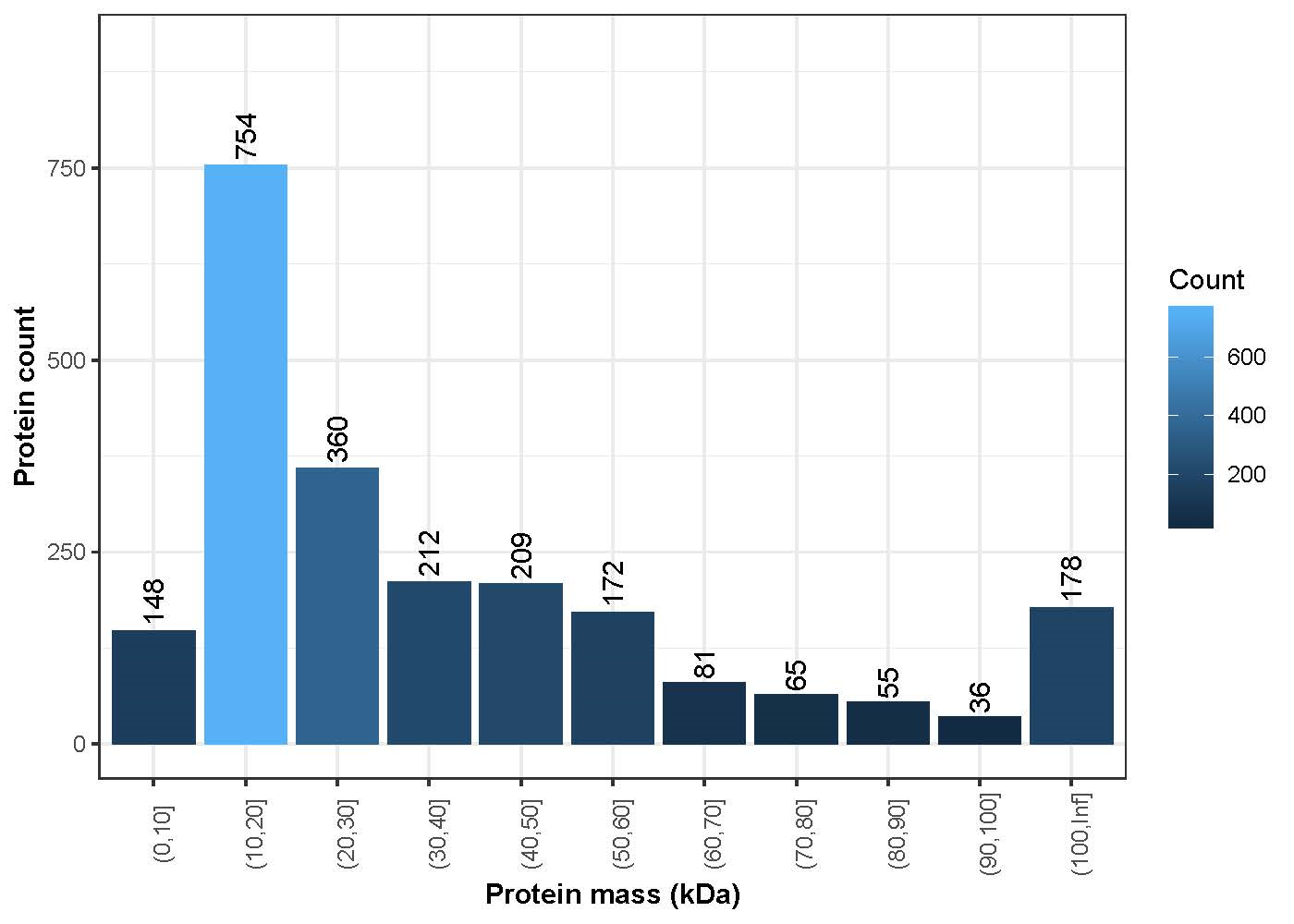


**Fig. 2.** Protein mass distribution. The X axis represents the molecular weights (kDa) of the identified proteins, and the Y axis represents the number of proteins.

**Table S1.** List of DEPs in patients with T2DM compared to control

| **Accession**  **number** | **Gene Name** | | **Description** | **Adjusted *P* value** | **FC** | **Expression** |
| --- | --- | --- | --- | --- | --- | --- |
| **O15049** | | N4BP3 | NEDD4-binding protein 3 | 0.04 | 1.72 | Up |
| **O75460** | | ERN1 | Serine/threonine-protein kinase/endoribonuclease IRE1 | 0.03 | 2.07 | Up |
| **P00915** | | CA1 | Carbonic anhydrase 1 | 0.003 | 4.87 | Up |
| **P00918** | | CA2 | Carbonic anhydrase 2 | 0.004 | 3.94 | Up |
| **P01160** | | NPPA | Natriuretic peptides | 0.026 | 3.10 | Up |
| **P02042** | | HBD | Hemoglobin subunit delta | 0.001 | 5.06 | Up |
| **P02533** | | KRT14 | Keratin, type I cytoskeletal 14 | 0.018 | 22.24 | Up |
| **P06703** | | S100A6 | Protein S100-A6 | 0.004 | 2.59 | Up |
| **P07738** | | BPGM | Bisphosphoglycerate mutase | 0.007 | 3.00 | Up |
| **P0CG47** | | UBB | Polyubiquitin-B | 0.029 | 1.96 | Up |
| **P10599** | | TXN | Thioredoxin | 0.012 | 2.03 | Up |
| **P14174** | | MIF | Macrophage migration inhibitory factor | 0.008 | 2.19 | Up |
| **P18065** | | IGFBP2 | Insulin-like growth factor-binding protein 2 | 0.024 | 4.05 | Up |
| **P26447** | | S100A4 | Protein S100-A4 | 0.071 | 2.43 | Up |
| **P30041** | | PRDX6 | Peroxiredoxin-6 | 0.026 | 2.60 | Up |
| **P30043** | | BLVRB | Flavin reductase (NADPH) | 0.004 | 4.213 | Up |
| **P30046** | | DDT | D-dopachrome decarboxylase | 0.016 | 2.08 | Up |
| **P30990** | | NTS | Neurotensin/neuromedin N | 0.009 | 1.88 | Up |
| **P32119** | | PRDX2 | Peroxiredoxin-2 | 0.002 | 4.57 | Up |
| **P35908** | | KRT2 | Keratin, type II cytoskeletal 2 epidermal | 0.013 | 2.44 | Up |
| **P68871** | | HBB | Hemoglobin subunit beta | 0.004 | 3.12 | Up |
| **Q06830** | | PRDX1 | Peroxiredoxin-1 | 0.035 | 2.64 | Up |
| **Q5K130** | | CLLU1-AS1 | Putative chronic lymphocytic leukemia up-regulated protein 1 opposite strand transcript protein | 0.028 | 3.88 | Up |
| **Q6E0U4** | | DMKN | Dermokine | 0.025 | 2.43 | Up |
| **Q76M96** | | CCDC80 | Coiled-coil domain-containing protein 80 | 0.036 | 2.35 | Up |
| **Q7KZ85** | | SUPT6H | Transcription elongation factor SPT6 | 0.017 | 3.18 | Up |
| **Q8NGJ1** | | OR4D6 | Olfactory receptor 4D6 | 0.006 | 1.92 | Up |
| **A0A1S5UZ39** | | HBA2 | Hemoglobin subunit alpha | 0.003 | 3.43 | Up |
| **B2M1S7** | | HBB | Beta-globin Showa Yakushiji variant (Fragment) | 0.026 | 3.66 | Up |
| **C8C504** | | HBB | Beta-globin | 0.006 | 4.16 | Up |
| **E9LUX2** | | HBA2 | Hemoglobin alpha-2 chain variant (Fragment) | 0.014 | 3.46 | Up |
| **E9PEW8** | | HBD | Hemoglobin subunit delta (Fragment) | 0.010 | 4.20 | Up |
| **G3V1N2** | | HBA2 | HCG1745306, isoform CRA_a | 0.002 | 5.44 | Up |
| **Q52MT0** | | HBB | Beta globin (Fragment) | 0.012 | 4.36 | Up |
| **Q5XTR9** | | HBD/HBB | Hemoglobin delta-beta fusion protein (Fragment) | 0.006 | 5.66 | Up |
| **Q9BWU5** | | HBB | Mutant hemoglobin beta chain (Fragment) | 0.004 | 4.94 | Up |
| **Q9GZL9** | | HBB | Beta-globin (Fragment) | 0.001 | 5.66 | Up |
| **Q9UNL6** | | HBG2 | Hemoglobin gamma-G (Fragment) | 0.032 | 4.56 | Up |
| **Q9UNU2** | | C4B | Complement protein C4B frameshift mutant (Fragment) | 0.045 | 1.63 | Up |
| **U3PXP0** | | HBA2 | Alpha globin chain (Fragment) | 0.005 | 4.48 | Up |
| **V9H1D9** | | - | Alpha globin | 0.004 | 4.43 | Up |
| **A0A075B6I4** | | IGLV10-54 | Immunoglobulin lambda variable 10-54 | 0.003 | 0.44 | Down |
| **A0A0B4J2H0** | | IGHV1-69D | Immunoglobulin heavy variable 1-69D | 0.043 | 0.41 | Down |
| **O14960** | | LECT2 | Leukocyte cell-derived chemotaxin-2 | 0.015 | 0.51 | Down |
| **O15078** | | CEP290 | Centrosomal protein of 290 kDa | 0.021 | 0.22 | Down |
| **O96006** | | ZBED1 | Zinc finger BED domain-containing protein 1 | 0.013 | 0.25 | Down |
| **P01023** | | A2M | Alpha-2-macroglobulin | 0.004 | 0.54 | Down |
| **P01602** | | IGKV1-5 | Immunoglobulin kappa variable 1-5 | 0.042 | 0.64 | Down |
| **P02765** | | AHSG | Alpha-2-HS-glycoprotein | 1.58E-05 | 0.64 | Down |
| **P11940** | | PABPC1 | Polyadenylate-binding protein 1 | 0.014 | 0.53 | Down |
| **P49454** | | CENPF | Centromere protein F | 0.003 | 0.48 | Down |
| **P62241** | | RPS8 | 40S ribosomal protein S8 | 0.048 | 0.49 | Down |
| **Q01523** | | DEFA5 | Defensin-5 | 0.007 | 0.49 | Down |
| **Q0VAK6** | | LMOD3 | Leiomodin-3 | 0.024 | 0.53 | Down |
| **Q16769** | | QPCT | Glutaminyl-peptide cyclotransferase | 0.002 | 0.51 | Down |
| **Q8N3L3** | | TXLNB | Beta-taxilin | 0.02 | 0.19 | Down |
| **Q96QR1** | | SCGB3A1 | Secretoglobin family 3A member 1 | 0.022 | 0.48 | Down |
| **Q9H0Y0** | | ATG10 | Ubiquitin-like-conjugating enzyme ATG10 | 0.002 | 0.57 | Down |
| **Q9HCJ2** | | LRRC4C | Leucine-rich repeat-containing protein 4C | 0.004 | 0.63 | Down |
| **Q9HCJ5** | | ZSWIM6 | Zinc finger SWIM domain-containing protein 6 | 0.036 | 0.56 | Down |
| **Q9NP71** | | MLXIPL | Carbohydrate-responsive element-binding protein | 0.006 | 0.10 | Down |
| **Q9NZP8** | | C1RL | Complement C1r subcomponent-like protein | 0.028 | 0.65 | Down |
| **Q9UNF0** | | PACSIN2 | Protein kinase C and casein kinase substrate in neurons protein 2 | 0.033 | 0.62 | Down |
| **Q9Y287** | | ITM2B | Integral membrane protein 2B | 0.018 | 0.65 | Down |
| **A0A068LN03** | | - | Ig heavy chain variable region (Fragment) | 0.011 | 0.46 | Down |
| **A0A120HF66** | | - | IBM-A1 heavy chain variable region (Fragment) | 0.003 | 0.41 | Down |
| **A0A120HG39** | | - | MS-A3 heavy chain variable region (Fragment) | 0.038 | 0.43 | Down |
| **A0A125U0U7** | | - | MS-C1 heavy chain variable region (Fragment) | 0.021 | 0.62 | Down |
| **A0A193CHR3** | | - | 10E8 heavy chain variable region (Fragment) | 0.002 | 0.47 | Down |
| **A0A1B0GUS7** | | UNC13B | Protein unc-13 homolog B | 0.002 | 0.49 | Down |
| **A0A1L2BU38** | | - | Anti-staphylococcal enterotoxin E heavy chain variable region (Fragment) | 0.012 | 0.26 | Down |
| **A0A1W2PQ80** | | IGLV10-54 | Immunoglobulin lambda variable 10-54 | 0.006 | 0.38 | Down |
| **A0A1W6IYJ1** | | - | N90-VRC38.09 heavy chain variable region (Fragment) | 0.019 | 0.62 | Down |
| **A0A2U8J8I9** | | IgH | Ig heavy chain variable region (Fragment) | 0.036 | 0.49 | Down |
| **A0A2U8J8J8** | | IgH | Ig heavy chain variable region (Fragment) | 0.014 | 0.27 | Down |
| **A0A2U8J8K9** | | IgH | Ig heavy chain variable region (Fragment) | 0.041 | 0.52 | Down |
| **A0A2U8J8R6** | | IgH | Ig heavy chain variable region (Fragment) | 0.031 | 0.41 | Down |
| **A0A2U8J959** | | IgH | Ig heavy chain variable region (Fragment) | 0.043 | 0.53 | Down |
| **A0A2Y9CYF7** | | IgH | Ig heavy chain variable region (Fragment) | 0.0002 | 0.52 | Down |
| **A2J1N9** | | - | Rheumatoid factor RF-ET12 (Fragment) | 0.038 | 0.45 | Down |
| **A2J423** | | - | Anti-MplscFv (Fragment) | 0.0002 | 0.48 | Down |
| **A2MYD1** | | V4-4 | V4-4 protein (Fragment) | 0.027 | 0.36 | Down |
| **A2N2F4** | | VK3 | VK3 protein (Fragment) | 0.047 | 0.61 | Down |
| **B2RNT9** | | UHRF1BP1 | UHRF1BP1 protein | 0.039 | 0.64 | Down |
| **B7Z4D1** | | - | cDNA FLJ52630 | 0.022 | 0.56 | Down |
| **Q6N095** | | DKFZp686K03196 | Uncharacterized protein | 0.004 | 0.50 | Down |
| **Q6VFQ6** | | HBB | Hemoglobin beta chain (Fragment) | 0.048 | 0.06 | Down |
| **Q7Z3Y5** | | VKA17 | Rearranged VKA17 V gene segment (Fragment) | 0.016 | 0.36 | Down |
| **S6BAM6** | | - | IgG H chain | 0.002 | 0.53 | Down |
| **V9HW34** | | HEL-213 | Epididymis luminal protein 213 | 0.0001 | 0.65 | Down |
